# 58201 What does team science look like across the CTSA Consortium? A qualitative analysis of the Great CTSA Team Science Contest results

**DOI:** 10.1017/cts.2021.581

**Published:** 2021-03-30

**Authors:** Clara Pelfrey, Ann Goldman, Deborah DiazGranados

**Affiliations:** 1 Case Western Reserve University; 2 George Washington University Milken Institute School of Public Health; 3 Virginia Commonwealth University

## Abstract

ABSTRACT IMPACT: This paper reveals the myriad techniques that CTSA hubs use to support, promote and expand team science including many ways to involve the community, students, scholars and other multidisciplinary scientists. OBJECTIVES/GOALS: The Great CTSA Team Science Contest (GTSC) was developed in the NCATS Workgroup on Institutional Readiness for Team Science to collect stories describing the many ways hubs were promoting and supporting team science across the CTSA consortium. METHODS/STUDY POPULATION: Our qualitative data analysis examined the different designs from a high level - namely we categorized how many of the stories were competitions for pilot funding, training programs on team science competencies, communication skills training, workshops for educating community collaborators about research and/or training investigators about community-based research, advancing promotion and tenure for team science, etc. We discuss specific examples of different designs and who they were intended to benefit. RESULTS/ANTICIPATED RESULTS: Launched in July 2018, the contest received 170 submissions from 45 unique CTSA hubs. Qualitative analysis addressed the following questions about team science: 1) Who or what group championed it? 2) Who benefitted or who were the intended recipients? 3) What was the desired outcome? (e.g. team science skills, communication skills, getting the community involved, fostering new collaborations, expanding capacity for team science, etc.) 4) What method(s) did they use? 5) What translational science stage was addressed? DISCUSSION/SIGNIFICANCE OF FINDINGS: This analysis includes examples of team science research, resources or interventions including successful team dynamics and knowledge integration. This paper reveals the myriad techniques that CTSA hubs use to support, promote and expand team science including involving the community, students, scholars and other multidisciplinary scientists.

